# A Journey From SARS-CoV-2 to COVID-19 and Beyond: A Comprehensive Insight of Epidemiology, Diagnosis, Pathogenesis, and Overview of the Progress into Its Therapeutic Management

**DOI:** 10.3389/fphar.2021.576448

**Published:** 2021-02-26

**Authors:** Muhammad Harris Shoaib, Farrukh Rafiq Ahmed, Muhammad Sikandar, Rabia Ismail Yousuf, Muhammad Talha Saleem

**Affiliations:** Department of Pharmaceutics, Faculty of Pharmacy and Pharmaceutical Sciences, University of Karachi, Karachi, Pakistan

**Keywords:** coronavirus, SARS-CoV-2, COVID-19, remdesivir, dexamethasone, hydroxychloroquine, azithromycin, vaccines

## Abstract

The 2019 novel coronavirus (2019-nCoV), commonly known as severe acute respiratory syndrome coronavirus 2 (SARS-CoV-2) or coronavirus disease 2019 (COVID-19), was first revealed in late 2019 in Wuhan city, Hubei province, China. It was subsequently spread globally and thereby declared as a pandemic by WHO in March 2020. The disease causes severe acute respiratory illness and is highly contagious due to the fast-onward transmission. As of the mid of November 2020, the disease has affected 220 countries with more than 16 million active cases and 1.3 million deaths worldwide. Males, pregnant women, the elderly, immunosuppressed patients, and those with underlying medical conditions are more vulnerable to the disease than the general healthy population. Unfortunately, no definite treatment is available. Although remdesivir as an antiviral had been approved for use in those above 12 years of age and 40 kg weight group, it has been observed to be ineffective in large-scale SOLIDARITY trials by WHO. Moreover, dexamethasone has been found to increase the recovery rate of ventilated patients; oxygen and inhaled nitric oxide as a vasodilator have been given emergency expanded access. In addition, more than 57 clinical trials are being conducted for the development of the vaccines on various platforms. Two vaccines were found to be significantly promising in phase III results. It is concluded that till the approval of a specific treatment or development of a vaccine against this deadly disease, the preventive measures should be followed strictly to reduce the spread of the disease.

## Introduction

### SARS-CoV-2 and COVID-19

**FIGURE 1:**
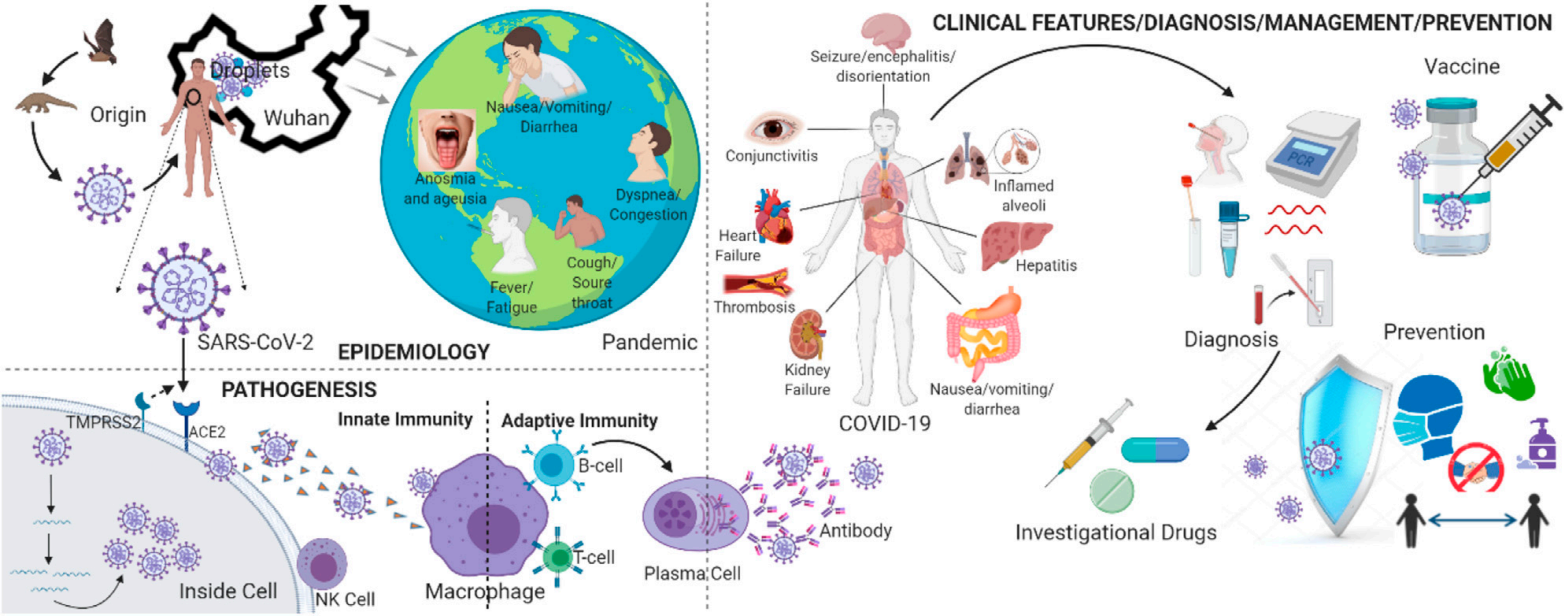
Schematic overview of SARS-CoV-2 epidemiology, pathogenesis and clinical features, diagnosis, management, and prevention [Figure created with BioRender, www.biorender.com].

Severe acute respiratory syndrome coronavirus 2, which is abbreviated as SARS-CoV-2, is a single-stranded RNA virus that belongs to the Coronaviridae family (subfamily: Coronavirinae) in the order Nidovirales*.* The consensus report after its phylogenetic analysis by Coronaviridae Study Group of International Committee on Taxonomy of Viruses has concluded that this virus belongs to a species group of similar coronaviruses called “Severe Acute Respiratory Syndrome-Related Viruses”. This particular virus has thus been recognized as “novel” in its phylogenetic character and is far more distinct than just strain and isolate of any previously known viruses (Gorbalenya et al., 2020). On January 3, 2020, the virus was first named 2019-nCoV (2019 novel coronavirus), and the disease was called novel coronavirus-infected pneumonia (NCIP) by the National Health Commission and China CDC after the revelation and analysis of the complete viral genome of the virus (Wenjie et al., 2020; Zhu et al., 2020). WHO has termed the infectious disease caused by SARS-CoV-2 as coronavirus disease 2019 (COVID-19) (WHO, 2020d). The collective symptoms due to the infection include a range of mild symptoms such as fever, dry cough, malaise, sore throat, fatigue, pain, and loss of taste or smell to a range of moderate symptoms predominantly including dyspnea, diarrhea, and pneumonia (Tay et al., 2020a; CDC, 2020). In critical situations, the patients were found to have been affected by dysfunctional immune response clinically identified as “cytokine release syndrome” and thrombosis, which often lead to fatal consequences (Merad and Martin, 2020). Since the initial report of the outbreak of the virus in Wuhan city, Hubei province, China, in December 2019, where a cluster of infections with pneumonia-like symptoms was reported, WHO declared COVID-19 a pandemic on March 11, 2020. Currently, it affects almost 220 countries across the world, with widely varying distribution of incidence and mortality among different geographies and countries. As of November 15, 2020, the total incidence of the infection stands at more than 57 million people diagnosed, with more than 1.3 million confirmed deaths reported globally (WHO, 2020a). A graphical presentation of the COVID 19 disease is presented in Figure 1.

### Morphology and Genetic Composition

**FIGURE 2:**
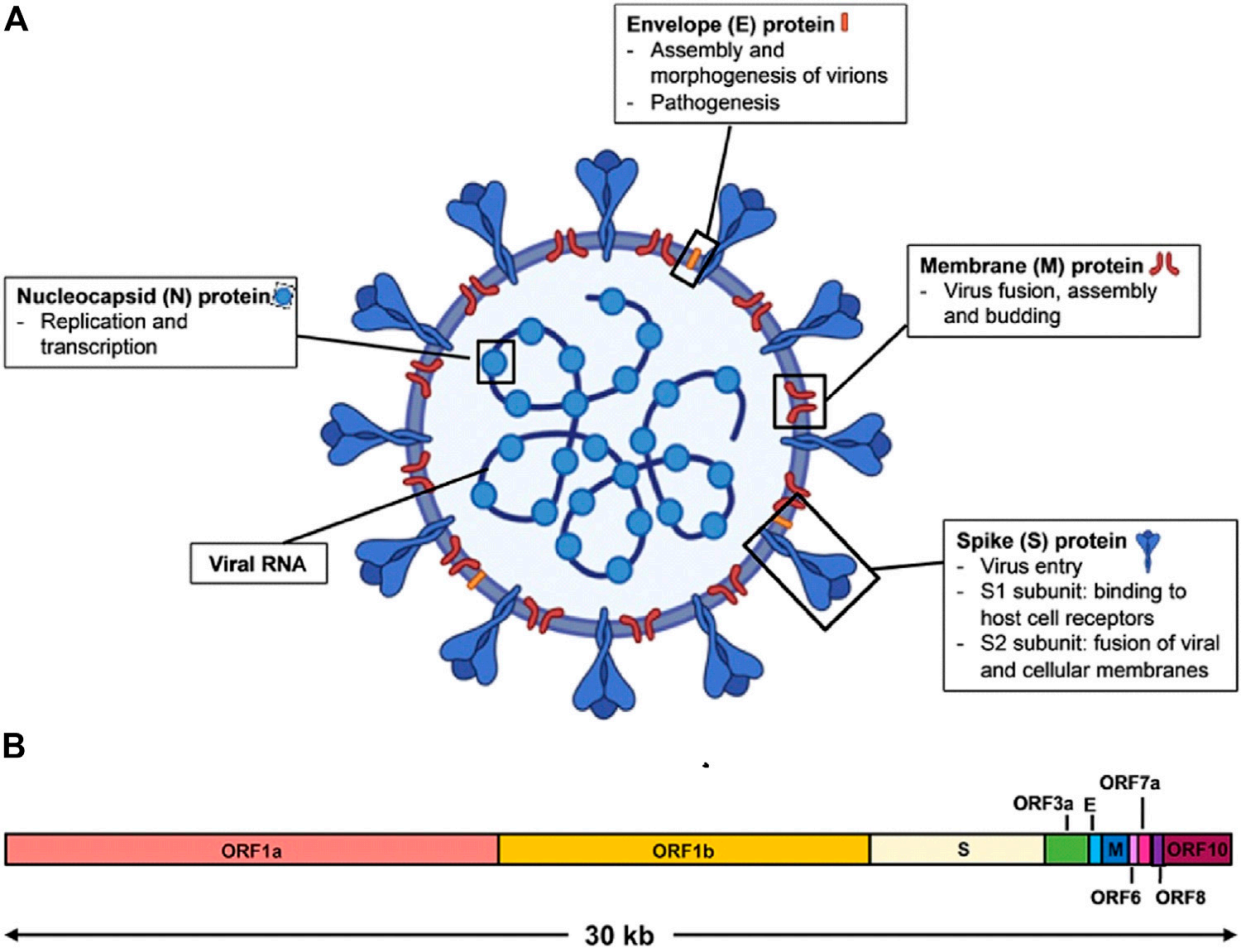
Schematic diagram of SARS-CoV-2 virus structure and genome organization. **(A)** The viral surface proteins, spike (S), envelope (E), and membrane (M) are embedded in a lipid bilayer. The single-stranded positive-sense viral RNA is associated with the nucleocapsid (N) protein. Diagram was created with BioRender. **(B)** The genome organization of SARS-CoV-2 viral RNA, which is adapted from GenBank accession number: MN908947, is characterized by sequence alignment against two representative members of the betacoronavirus genus. The entire genome sequence is ∼30 kb long [reproduced with permission] (Lee et al., 2020b).

The SARS-CoV-2 is a spherical-shaped virus with irregular crown-like projections on its surface. These crowns are surrounded by several types of functional proteins submerged and protruding from them. It is enveloped with a positive-sense single-stranded RNA genome with an approximate size of 30 kilobases (Zhao et al., 2020). In terms of its size, it has an overall large diameter in a size range of 75–160 nm (Guobao et al., 2007). The genome of SARS-CoV-2 consists of 14 open reading frames (ORFs) that encode 27 proteins, 15 nonstructural proteins that are important for viral replication, and four structural proteins named spike (S), envelope (E), membrane (M), and nucleocapsid (N) along with accessory proteins (Malik et al., 2020; Wu A. et al., 2020) (see Figure 2). The studies have indicated its similarity to Bat-SARS-like coronavirus, SARS-CoV, and MERS-CoV (Zhou P. et al., 2020; Chen Y. et al., 2020).

### Mechanism of Cell Entry and Life Cycle of the Virus

The virus enters the host’s cell through angiotensin-converting enzyme 2 (ACE2) receptors present on the cell membrane of the cells of several tissues, particularly of the lower respiratory tract (LRT), heart, kidneys, and gastrointestinal tract (GIT) (Imai et al., 2010; Hoffmann et al., 2020; Wan et al., 2020).

The entry is also facilitated by TMPRSS2 protease or endosomal cathepsin L present on host’s cells. The viral S protein consists of S1 and S2 subunits. The S1 binds ACE2 receptors through the RBD region, while S2 and TMPRSS2 or cathepsin L complex promote membrane fusion between the virus and the host cell. The entry is followed by the release of viral RNA, translation of ORF, production of nonstructured proteins, and formation of viral replication transcriptase complex. The complex initiates genome replication and subgenomic transcription. The viral structural proteins (S, E, M, and N) are encoded, including certain accessory proteins. Afterward, translation proteins are assembled at the endoplasmic-reticulum-Golgi intermediate compartment (ERGIC). Here, the S protein may also be modified by furin. The viral particles are thereby released from the host’s cell through exocytosis (Hoffmann et al., 2020; Tang T. et al., 2020; Shereen et al., 2020; Su and Wu, 2020).

**FIGURE 3:**
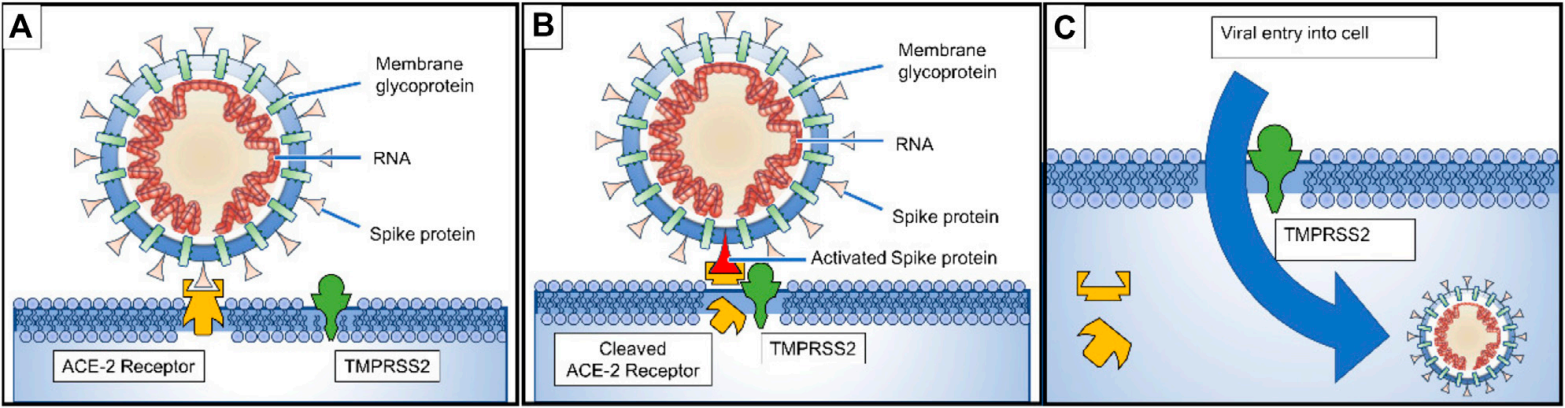
**(A)** Spike proteins on the surface of the coronavirus bind to angiotensin-converting enzyme 2 (ACE2) receptors on the surface of the target cell; **(B)** The type II transmembrane serine protease (TMPRSS2) binds to and cleaves the ACE2 receptor. In the process, the spike protein is activated; **(C)** cleaved ACE2 and activated spike protein facilitate viral entry. TMPRSS2 expression increases cellular uptake of the coronavirus (Lee et al., 2020).

This is the same mechanism as that observed previously for the SARS-CoV virus (Imai et al., 2010; Hoffmann et al., 2020; Wan et al., 2020). Some studies have found that the ACE2 receptor affinity of SARS-CoV-2 is more efficient than that of SARS-CoV(2003) but less efficient than its 2002 strain (Guobao et al., 2007; Wu A. et al., 2020) (see Figure 3). It is believed that any mutation on the receptor-binding domain (RBD) of S protein could make the virus more pathogenic. However, some mutations other than the receptor interaction sites in RBD of S protein have been discovered, but the role of such mutations in its pathogenicity is still not clear (Wu A. et al., 2020; Wan et al., 2020).

## Epidemiology

### Origin

The novel coronavirus (nCoV), which has been later named SARS-CoV-2, was first reported to spread among contacts in the Huanan seafood wholesale market in Wuhan city, Hubei province, China (Wang et al., 2020b). The isolated agent was later identified as the seventh species of the coronavirus family to have caused infectious conditions in humans (Tang X. et al., 2020). It has been strongly believed that the species originated from *Rhinolophus affinis* (horseshoe bats, 96% identical genome with RaTG13 coronavirus species found in the host) with likely zoonotic spillover in *Manis javanica* intermediary host (Malayan pangolins, identification of strong similarity in six places of the RBDs of the virus with species in the hosts). This assessment substantiates the argument of its natural selection in humans either before or after zoonotic spillovers from intermediary hosts (Andersen et al., 2020).

### Geographical Epidemiology, Ethnicity, and Culture

The SARS-CoV-2 virus has affected over 220 countries as of November 15, 2020. The epicenters of the pandemic are currently centered in the Americas (the United States and Central American and South American countries) and Europe, where the incidence is reported to be more than 24 million and 16 million cases, respectively (WHO, 2020a). In terms of mortality, United States, Mexico, Brazil, Columbia, and Peru are the worst affected countries in the Americas, whereas the United Kingdom, Spain, Italy, and France depict a similarly grim picture in Europe. India, Iran, and Russia are also among those countries that are badly affected globally (WHO, 2020a). In the United States, there have been approximately 11.5 million cases (highest incidence) reported with mortality of over 250,000. This is the highest mortality of any country reported so far. In terms of country-wise mortality figures, United States is followed by Brazil (∼168,000), Mexico (∼100,000), Argentina (∼37,000), Columbia (∼35,000), and Peru (∼34,000). Among the various European countries that have been hit badly by the pandemic, the highest mortality is documented in the United Kingdom (∼54,000), followed by Italy (∼48,000), France (∼47,000), and Spain (∼42,000). In Asia, India leads the mortality figures with more than ∼133,000 deaths attributed to COVID-19, followed by Iran with ∼44,000 deaths and Russia with ∼35,000 deaths (WHO, 2020e). Recently, many countries have seen a significant spike or surge in the number of new cases since the start of September 2020. This is especially true for many European countries where collectively ∼270,000 cases are now being reported each day in Europe against a modest value of ∼30,000 back in late August. Similarly, these figures are also being reported for the Americas against the lowest values ∼65,000 in August (WHO, 2020e). This is being referred to as the “second wave” of new infections, especially pertinent to European countries, where after significant depression in the number of new cases in recent 3–4 months (due to government measures), the recent lifting of lockdowns has led to a huge spike of new cases (Looi, 2020). Governments have therefore implemented strong measures equivalent to initial measures taken during the first wave of infections.

There is wide intercontinental variability in the spreading of COVID-19, and to assess the regional population data, individual expression of transmembrane protease serine 2 (TMPRSS2), which allows cellular uptake of the S protein, may be a determinant of SARS-CoV-2 regional infection susceptibility. Four variants of TMPRSS2 were evaluated in the local population of Africa, America, Europe, and Asia (China, Japan, and Taiwan), and the frequencies of variant alleles with high TMPRSS 2 expression in lungs were reported to be highest in the European and American population and lowest in the Asian population. Similarly, phylogenic analysis of time to the most recent common ancestor (TMRCA) was performed for SARS-CoV-2, dating to November 12, 2019, which also matched the epidemiological records of the disease. The non-Asian (Africa and Europe) outbreak of the disease has been associated with the reported reason of subhaplogroup A2 originated in Europe from Asian ancestor, where haplogroup A is regarded as an ancestral node (Gómez-Carballa et al., 2020). The emergence of COVID-19 was late in South America. The first case was reported on the 25th of February 2020 in Brazil, after which the entire country went into strict lockdown within few weeks. More than 65,000 cases were reported on the 14th of April, and Ecuador was the one found to be badly affected. The high incidence of COVID-19 was found in the state of Ceara, and most of the cases were originated from Fortaleza, the capital. The exponential rise in COVID-19 cases also led to the need for epidemiological surveillance and consistent analysis (Burki, 2020). A bibliometric study was also performed to evaluate the current trend in research on COVID-19 conducted in Latin America, and Aldeota, Cais do Porto, Centro, Edson Queiroz, and Cambeba were found to be the neighborhoods with the highest propensity for COVID-19 (Braga et al., 2020).

Ethnic factor is also a significant factor determining vulnerability among different racial groups. The susceptibility of COVID-19 among different races was reported in the following order: Black (Central and Western Africa) > Asian (Bangladesh > Indian ≥ Chinese ≥ Pakistani) > mixed ethnic groups (Epi cell, 2020). Similarly, culture plays a vital role in determining the attribution to disease, help-seeking behavior, and community acceptance to comply with measures and interventions to counter pandemic spread. Cultural norms greatly influence the failure or success of the strategies derived for the containment of the COVID-19 outbreak. They may augment the community response to volunteering efforts and making social distancing and lockdown an easier task (Mckee, 2020). For example, some Asian countries like Japan, Vietnam, and Taiwan implemented stringent lockdown at the beginning of the outbreak, overlooking their national economic damage. However, for the United States of America, the primary concern was their economy, and American authorities neglected the severity of the COVID-19 outbreak (Huynh, 2020). In addition, 1.76 million people might have been saved in the United States if strict social distancing was practiced. The Russian approach was more different, comprising penalties against the violation of the governmental instructions, whereas India and the Philippines were more stringent and were reported to arrest people not following social distancing practice (Greenstone and Nigam, 2020). Fetzer et al. (2020), studied the heterogeneous behavior of 51 countries for following social distancing and reported that Peru strictly followed the “staying at home” policy and ranked second among the countries with the highest percentage of residentials (Fetzer et al., 2020). The Asian countries applied a punitive approach to social distancing as their strict cultures, while the European countries are likely to be lenient in forcing people to stay at home. It has also been observed that countries with higher “Uncertainty Avoidance Index (UAI),” exhibited a lower proportion of public gathering. UAI shows that people in a society are in fear of unknown, uncertain, and unstructured situations; this can influence cultural perception and decision power (Huynh, 2020). The perceptions of different communities also affect the local medicinal practices; in a study performed in India, 48% of participants favored eating garlic in prophylaxis against COVID-19 (Vadivu and Annamuthu, 2014). But there can also be a sense of truculence and false hope that may drive some communities out of isolation due to a high degree of hubris. For example, in India, some organizations promoted people to take a bath in cow dung to defeat the SARS-CoV-2 (Theinterpreter, 2020).

### Demographic Distribution

In terms of the distribution of incidence rates among different age and racial groups, the virus seems to be very discriminative. The data of 10 European countries on the distribution of COVID-19 cases by gender and age revealed that females of working age outnumber infected males. However, the rate in females declines at the retirement age of 60–69, which results in a crossover among males with COVID-19, so the vulnerable age group for a male is 70–79 and that of the female is 20–29, especially those bearing health and care–related occupation profiles (Sobotka et al., 2020). Moreover, it has been found that pregnant women are more vulnerable to COVID-19 infection, with a higher rate of death being associated with physiological changes during pregnancy, such as an increase in heart rate, a decrease in lung capacity, and a higher risk of thromboembolic disease (Centers for Disease Control and Prevention (CDC), 2020b). Across the globe, one pattern is very persistently observed; that is, infected males are more likely to die than females, despite all the uncertainties and dubitation (Zhou F. et al., 2020; Jordan et al., 2020; Lawton, 2020). Globally, in terms of the incidence of symptomatic incidence and mortality, elderly patients, especially with underlying conditions, are identified as the most vulnerable group (Kang and Jung, 2020). In New York state for the two oldest age groups, 65–74 years old and 75 and above, the weekly calculated infection fatality risk (IFR) was more significant than that of the younger age (0.0097% for <25 years and 0.12% for 25–44 years) group and was reported to be 6.7% and 19.1%, respectively (Yang W. et al., 2020). The cumulative infection rate across the globe is the lowest, i.e., below 1 per 1,000 among children and adolescents. The rates were found to be lowest in Spain, 0.6% (age, 0–14), and highest in Czechia, 9.2%. Comparatively stable infection rates were reported in Portugal at the ages of 20–59, but the irregular profile was exhibited in Czechia and Germany; however, a steeper rise in infection rate with age was observed in England (Sobotka et al., 2020).

Empirically, older people with comorbidities, residing in nursing homes, are at the highest risk of adverse outcomes and mortality during the running phases of a pandemic. Moreover, behavioral problems, cognitive disorders, and functional impairment may synergize the threat posed to nursing homes (Fallon et al., 2020). In Washington, about two-thirds of the residents were reported to be infected within a period of 3 weeks only at the death rate of 33%, along with 50 staff members and 16 visitors infected (Mcmichael et al., 2020). A significant number of deaths reported in Spain have been associated with nursing home residents. Many authorities are not including deaths at nursing homes from the total COVID-19 death toll (Wang et al., 2020a).

### Mode of Transmission

SARS-CoV-2 has been found to transmit primarily through respiratory droplets (5–10 µm) and physical contact with contaminated matter. It is believed that the virus could also be transferred through airborne mechanisms (aerosols) where the virus gets trapped in particles (<5 µm) for an extended period of time and can be transmitted through distances of over 1 m (WHO, 2020c; Liu et al., 2020b; Morawska and Cao, 2020). Such instances are likely in closed spaces such as saturated ventilation systems or proximities where the patients with fluids of high virus loads are in contact with the susceptible individuals, such as exposure of healthcare workers and medics during intubation procedures and noninvasive positive pressure ventilation (Liu et al., 2020b). There have been reports that SARS-CoV-2 is also present in the fecal matter of the patients and can infect the gastrointestinal tract (Lamers et al., 2020). However, the scientific evidence for confirmed transmission through the fecal-oral route is missing (Xu Y. et al., 2020). Furthermore, there have been instances where the virus has been found to transfer through another intermediary host such as domesticated cats (human-cat-human), raising significant concerns of additional factors that could aid in the spread of the virus (Shi J. et al., 2020; Halfmann et al., 2020; Mallapaty, 2020).

In terms of epidemic modeling, the mean reported “reproduction number” (*R0*) for the current first wave of the pandemic has been estimated around 3.28 with a median value of 2.79, which is surprisingly very high compared to the estimates of 1.25–2.5 given by WHO at the beginning of the epidemic (Liu et al., 2020a). Moreover, this estimate is also higher than ∼2 reported for SARS-CoV-1. The surprising element behind the unprecedented spread of this coronavirus is its capability of transmission presymptomatically (∼48%, cases that show symptoms afterward) and asymptomatically (∼10%, cases where the person does not show any symptoms). The symptomatic cases are reported to be around 38% of the total transmissions (Ferretti et al., 2020). It must also be mentioned here that citing some recent studies, WHO, in its interim guidance and public press briefing, has made claims that asymptomatic transfer is unlikely with a wide level of interest in the announcement by governments around the world in favor of reopening the economies; however, due to significant criticism from public health experts around the world, WHO has since changed the stance and maintained that the matter is not yet close to a verdict and even if there is weak evidence of asymptomatic transfer, there is still a chance of its spread. As far as the current spread is concerned, WHO has categorized the transmission within countries as either “sporadic,” “cluster-based,” or “community-based” transmissions and thus with the current trend of data, Saudi Arabia, Somalia, Yemen, and Magnolia are classified as “sporadic transmission” areas, whereas countries such as China, Pakistan, India, Australia, Russia, Germany, Italy and Portugal are being classified as “cluster-based transmission” areas. The remaining areas are largely classified as “community-based transmission” areas, including the Americas, Africa, and remaining countries of Europe.

### Precipitating Factors Influencing the Transmission

Wide varieties of factors have been identified to influence and affect the transmission rate of SARS-CoV-2 and the severity of COVID-19 among humans. Some of these factors are related to social behavior, while others are identified as physical and environmental conditions. According to a detailed study investigating the interrelationship of various factors influencing the virulence of the COVID-19, the primary factors to limit the spread of COVID-19 are social distancing and community sense of mitigation measures recommended by WHO such as personal hygiene and mandatory wearing of face masks, especially in closed spaces (Lakshmi Priyadarsini and Suresh, 2020). Conversely, in terms of physical conditions, lower air temperature (∼22°C) and lower relative humidity (40–60% RH), turbulent airflow patterns in packed areas, and closed-circuit ventilation have all been reported to participate in the spread of contaminated aerosols and thus are likely participants in the increased rate of transmission. Primary physical factors related to environmental conditions such as air temperature, relative humidity, and UV light exposure have been previously studied in detail for the SARS-CoV-1 virus, with a significant loss of virulence observed at a temperature of 38°C and >95% RH (Chan et al., 2011; Kowalski et al., 2020). Although there are few such studies on SARS-CoV-2, the same effects have been observed related to temperature and relative humidity for this virus, thereby impacting its spread (Bannister-Tyrrell et al., 2020). The virus has been found to be stable over a wide range of pH (3–10) at room temperature (Chin et al., 2020). The stability studies on different surfaces have identified that the viral titer was undetectable from printing or tissue paper after 3 h incubation and 2 h on wood and cloth but could last for 4–7 days on other surfaces. Furthermore, it has been found to be susceptible to typical disinfectants such as hydrogen peroxide, sodium hypochlorite, and hand soaps (Chin et al., 2020).

Another significant factor related to demographics is the age bracket of exposed individuals (higher age groups are more susceptible), as it is continuously reported as a significant predisposing factor for increased mortality and morbidity due to COVID-19 (Lakshmi Priyadarsini and Suresh, 2020). The other major precipitating factor that has been noted to contribute to the severity of COVID-19 is underlying medical conditions, such as hypertension, diabetes, asthma, renal disease, and other respiratory conditions such as COPD (Lakshmi Priyadarsini and Suresh, 2020). It has been suggested that the higher baseline levels of IL-6 and other inflammatory cytokines may be the probable reason for severe infection in these conditions (Tay et al., 2020b; Lakshmi Priyadarsini and Suresh, 2020). However, the anticipated response is not observed in inflammatory arthritis patients, even with raised levels of IL-6 (Schett et al., 2020). In addition, immune-compromised patients and patients on immunomodulators are also likely to exhibit a quite grim picture of COVID-19 (Monti et al., 2020). In a study, 58 patients with multiple myeloma (MM) receiving different immunomodulators diagnosed with COVID-19 showed a mortality rate of 24%. Therefore, early intervention in immunomodulatory therapies and strict adherence to the safety measures are recommended to encounter the future outbreak of COVID-19 (Wang B. et al., 2020).

Furthermore, one of the most significant socioenvironmental factors affecting the rate of spread is the population density. This factor alone can significantly contribute to the major wave of large-scale epidemics observed in localities like New York, New Jersey, and Indian Slums Metropolitan areas (Corburn et al., 2020; Gonzalez-Reiche et al., 2020; Mishra et al., 2020). Similarly, the transfer rate and infection reproduction number (*R* ^*0*^) were calculated to be four times higher than those in the initial spread in Wuhan in Diamond Princess (a cruise ship affected by SARS-CoV-2 (Rocklöv and Sjödin, 2020).

### Global Measures in Response to Pandemic

Since the first identification of the spread of this virus in the Wuhan city of Hubei province China, governments and policy advising agencies have been advocating for various containment and mitigation strategies to minimize the spread and flatten the pandemic curve to avoid crippling consequences on the healthcare systems due to out-of-capacity inflow of critical cases that may result in higher mortality. Four key response measures have been suggested by the OECD to the governments worldwide in light of the scientific evidence established from earlier and current pandemics. These include 1) large-scale surveillance, monitoring, and detection through centralized epidemiological and disaster management centers; 2) prevention of the spread in the community by means of social distancing measures and smart and complete lockdowns wherever necessary; 3) clinical management of cases by means of the best available scientific evidence and practices; 4) maintaining essential services to ensure the smooth running of the system and avoiding any other potential catastrophe (OECD, 2020). A mix of various containment and mitigation strategies well suited for the given country and region is advocated. In this regard, the United Nations had advised implementing contact tracing and hotspot mapping strategy to mitigate the spread for developing countries. This is especially true for developing countries such as Bangladesh, Myanmar, and India, where the economic consequences of complete lockdown are becoming disastrous (UN-DGC, 2020).

After a nearly complete global shutdown, economists are ringing alarms of the unprecedented recession of the 21st century, which is already devastating news for the developing economies, owing to their large informal sectors. The projected median decline in GDP is already threatening for many economies and may likely dip down further below the projections (Fernandes, 2020).

The measures to circumvent the spread of this virus based on manual contact tracing are insufficient and thus, along with the advocated measures of social distancing, hygiene, manual tracing of contacts, quarantine, and lockdowns, the use of digital applications, where the contacts are automatically alarmed of any potential transmission with a known case, is touted to be a major driving factor to control the spread. This strategy has been in various ways successful in countries such as South Korea if implemented with transparency and integrity to secure the public data (Ahn, 2020). Moreover, the strict implementation strategy and early timing of enforcing social distancing measures across many countries have resulted in a very contrasting and significant consequence in terms of reduction of *“R*” value of the spread and total fatalities and incidence of the infections (Ketchell, 2020).

## Diagnostic and Monitoring Tools

SARS-CoV-2 genetic material is reported to be successfully detected through throat swabs and the upper and lower respiratory tract, blood, stool, or urine samples (Pan Y. et al., 2020; Chen W. et al., 2020; Yan et al., 2020). Several methods have been introduced for the detection of SARS-CoV-2. However, the collection of various samples from different sites and the utilization of multiple techniques is usually recommended to avoid false results related to the use of a single sample or method (Wang et al., 2020b). Furthermore, the application of positive, negative, and inhibition controls is also recommended to assure quality diagnosis (Yan et al., 2020).

### Nucleic Acid Amplification Tests

#### PCR and Real-Time PCR

PCR and RT-PCR are considered important molecular biology techniques, first introduced in the 1980s. The techniques are based on the amplification and detection of a particular gene (Panawala, 2017). The amplification of genetic material is beneficial for obtaining the satisfying quantity of specimens required for a laboratory study. Both PCR and RT-PCR involve the utilization of certain enzymes. PCR uses a DNA template, whereas RT-PCR uses RNA (Panawala, 2017). Several RT-PCR-based test kits have also been developed (Li X. et al., 2020).

##### Sensitivity and Specificity

The techniques are considered highly sensitive, highly specific, and reliable for the detection of CoVs (Shen M. et al., 2020). However, these methods were observed to be commonly used for SARS-CoV-2 detection (Li X. et al., 2020). Yet, the procedures are claimed to be time-consuming and expensive that require costly reagents or equipment. The absence of safe and stable EPC (external positive controls), as available for SARS-CoV-1, is another severe problem in the detection of SARS-CoV-2 (Shen M.et al., 2020). Furthermore, adequate sampling, proper handling of the sample, and sufficient genetic material are recommended for a reliable PCR-based report (Lee et al., 2020). Additionally, RT-PCR is recommended over PCR due to its superior sensitivity (Shen M. et al., 2020).

Several rapid diagnosis kits that have been developed as per WHO standards are claimed to be 95% accurate against SARS-CoV-2. An RT-PCR-based test kit has also been introduced by the Centers for Disease Control and Prevention (CDC) CDC (2020b). Rapid nucleic acid diagnostic papers have also been invented, which provide a rapid detection facility of only 3 min with unaided eye observation (Jin Y. et al., 2020). Furthermore, the kits are only limited to upper and lower respiratory tract specimens. However, FDA has recently authorized the first RT-PCR-based LabCorp COVID-19 kit with a home collection option (FDA, 2020b).

#### Loop-Mediated Isothermal Amplification

LAMP is known as an ultrasensitive novel isothermal nucleic acid amplification–based method. It has been claimed to be capable of detecting even a small quantity of biomaterial within an hour and without the need for expensive reagents or equipment.

##### Sensitivity and Specificity

Its sensitivity and detection rate against coronaviruses have been found to be similar to those of PCR-based methods. However, the technique requires a high temperature, usually 65 °C, which restricts its application (Shen M. et al., 2020).

#### Microarray

The microarray technique has been widely used for the detection of coronaviruses. In this method, the virus RNA is used to produce cDNA, labeled with a specific probe through reverse transcriptase followed by subsequent detection of that specific probe. The method offers a low cost with sensitivity equal to that of RT-PCR. Moreover, portable microarray chips with adequate detection limits have also been introduced (Shen M. et al., 2020).

#### Specific High-Sensitivity Enzymatic Reporter Unlocking

This method is based on RNA-targeting CRISPR (Clustered Regularly Interspaced Short Palindromic Repeats) related enzyme Cas13. Cas13 has been combined with LAMP to detect DNA or RNA molecules.

##### Sensitivity and Specificity

The method has been shown to be quick, portable, and highly sensitive for nucleic acid detection (Shen M. et al., 2020; Udugama et al., 2020).

### Radiological Examination

Although the nucleic acid amplification test has been widely recommended for the detection of SARS-CoV-2, its false reports could not be overlooked that may result in a false diagnosis and other severe consequences (Li X. et al., 2020). Consequently, the CT (chest radiography) scan has become a reliable method for the diagnosis of COVID-19 in clinical practice (Jin Y. et al., 2020). The scan images of almost all COVID-19 cases indicate the same features, particularly bilateral pulmonary parenchymal ground-glass opacification and consolidative pulmonary opacities, that have been observed in nearly 60 to 77 percent of cases (Forouzesh et al., 2020). At the same time, it has been observed that patients with negative nucleic acid amplification tests may show positive chest CT scan findings. However, a repeated nucleic acid amplification test is suggested for the final remarks (Forouzesh et al., 2020). Artificial Intelligence (AI) technology has also been used for accurate and instant interpretation of CT images (Jin Y. et al., 2020; Mak, 2020).

#### Limitations

Some disadvantages of CT imaging have also been reported, particularly nonselectivity and hysteresis of irregular imaging (Li X. et al., 2020). Moreover, prevention from frequent exposure to radiation, especially for pregnant women and children, is strongly recommended (Forouzesh et al., 2020).

### Serological Tests

Acute serological responses have been identified in COVID-19 patients (Zhou P. et al., 2020). The serological tests are considered alternative to the nucleic acid test and CT imaging. For this purpose, several colloidal gold immunochromatography assays and other related techniques, kits, and detection methods have been applied and established (Jin Y. et al., 2020; Lee et al., 2020; Li X. et al., 2020; Rashid et al., 2020). The techniques generally target coronavirus immunogenic proteins (S, N, E, and M) and RBD to detect the presence of SARS-CoV-2 related antibodies (Mcintosh et al., 2020). The IgG levels are reported to be usually increased as the IgM levels start decreasing during viral infection (Rashid et al., 2020). IgM antibodies have been detected successfully during the early phases of infection, usually within 3 days, and are claimed to be present even after a month. Similarly, SARS-CoV-2 specific IgG antibodies have been reported to be detected after 4 days of infection period with a peak level after 2 weeks. It has been shown that their levels are related to disease severity; a higher level of both antibodies indicates greater severity of the infection. Researchers have also suggested IgA detection for SARS-CoV-2 diagnosis that is related to mucosal immunity usually activated in COVID-19 patients. However, it is considered less specific than IgM and IgG (Lee et al., 2020).

A list of other serological markers has been reported for the prediction of infection severity and prognosis of the disease in patients suffering from COVID-19. Some of these include an examination of interleukins (IL) levels, particularly IL-6, IL-10, and IL-2R, ESR (Erythrocyte Sedimentation Rate), CBC (Complete Blood Count), PT (Prothrombin Time), and levels of liver, kidney, heart, and other related enzymes (Forouzesh et al., 2020).

#### Limitations

Although serological tests are regarded as fast, powerful, and easy to conduct, it has been noted that the antibodies' response develops after several days of infection. The CDC does not recommend these tests for the diagnosis of current COVID-19 disease. Moreover, only 70% of their sensitivity is reported even after 4–6 days of infection (Centers for Disease Control and Prevention (CDC), 2020a; Wang et al., 2020b). The antibodies, IgM, and IgG, against SARS-CoV-2, have been observed to increase progressively with infection (Lee et al., 2020). Thereby, early diagnosis of infection is not possible and may lead to false-negative reports. It has also been reported that a large population has already been exposed to other human coronaviruses, and thus the false-positive response is commonly observed due to a high level of SARS-CoV-2 similarity to other coronaviruses. Therefore, the utilization of multiple serological approaches is recommended for a true report (Lee et al., 2020).

## Coronavirus Disease 2019

### Immune System

The immune system works as a defense system and plays a key role in the prevention of pathogenic attacks throughout the body; however, uncontrolled or impaired immune response may result in harmful tissue damage (Cao, 2020; Li G. et al., 2020). Overwhelming of the inflammatory response is considered to be initiated as a result of the antagonism effect of interferon by SARS-CoV-2 to promote its replication inside the cell (Tay et al., 2020a). Interferon (IFN) response is considered directly related to viral load. An increase in type 1 IFN response causes decreased viral load and vice versa. It has been observed that a decrease in total T cell count causes a declined function of these cells in COVID-19 patients (Diao et al., 2020). However, increased levels of cytokines such as interleukins (IL-6, IL-1β, IL-2, IL-8, and IL-17), granulocytes like granulocyte colony-stimulating factor (G-CSF), granulocyte-macrophage colony-stimulating factor (GM- CSF), interferon gamma-induced protein 10 (IP10), monocyte chemoattractant protein-1 (MCP1), macrophage inflammatory proteins-1 alpha (MIPα), and tumor necrosis factor (TNF) along with C-reactive proteins, D-dimers, and ferritin are reported in COVID-19 infection (Cao and Li, 2020; Xu Z. et al., 2020; Huang et al., 2020). Cytokines are responsible for shock and severe tissue damage to different organs, and slow healing of lungs is observed in patients with elevated IL-6 levels (Wang et al., 2020b). Another unique characteristic of hypercoagulation has also been commonly noticed in serious COVID-19 patients (Tang et al., 2020b; Merad and Martin, 2020). The cytokine storm and sepsis are considered the primary cause of death in about 28% of severe cases of COVID-19 (Zhang B. et al., 2020). But these immunological changes are often restored, particularly in mild to medium cases. Simultaneously, individuals with robust immunity and without comorbidities may successfully eliminate the virus before the exacerbation of immune overreaction (Cao and Li, 2020).

### Organs Involvement

The organs that have been confirmed clinically to be involved in the COVID-19 infection include the eye, nervous, digestive, respiratory, circulatory, and urinary systems (Wang L.-S. et al., 2020). Although the lungs are the primary target of COVID-19 infection, it can attack or damage almost all body organs, particularly the heart, blood vessels, kidneys, intestines, and brain. The cells of these organs are rich in ACE2 receptors that are essentially required for the virus entry into the cells (Cao and Li, 2020; Cyranoski, 2020; Ky and Mann, 2020; Wadman et al., 2020; Chris Baraniuk, 2020, April 29).

#### Nasal Passage

The cells of the nose and throat are rich in ACE2 receptors providing an adequate environment for the virus where it starts replication. This is an asymptomatic phase, but a person could be the carrier of this deadly virus to another person (Peiris et al., 2003; Zou et al., 2020; Chris Baraniuk, 2020). The viral load of SARS-CoV-2 has been found higher in the nose than throat, unlike SARS-CoV-1, which is the probable reason for its rapid spread through respiratory droplets on close person-to-person contact (Chavez et al., 2020; Zou et al., 2020). The other sources of its spread could be the air contaminated with viral load from cough or sneeze of an infected person, touching or shaking hands, touching the mouth, nose, or eye after viral exposure, or less frequently through the fecal-oral route(Joseph and Ashkan, 2020).

It has been observed that in some cases, viruses may bypass the throat cells and enter into the lungs directly and may cause acute pneumonia without developing mild symptoms related to the throat, including cough and low-grade fever. (Cyranoski, 2020; Wadman et al., 2020).

#### Lungs

The lungs are considered as the main battle area. The alveoli of the lungs are rich in ACE2 receptors (Wadman et al., 2020; Chris Baraniuk, 2020, April 29). The virus attacks epithelial cells of the lungs and causes Diffuse Alveolar Damage (DAD), resulting in respiratory failure in some patients (Gu and Korteweg, 2007; Schaefer et al., 2020). The WBCs (White Blood Cells), dead cells, mucous, and pus or fluid together in alveoli after the virus attack causes ARDS (Acute Respiratory Distress Syndrome) with parallel symptoms of pneumonia-like fever, cough, and difficulty in breathing, resulting in hypoxemia (Tay et al., 2020a; Xu Z. et al., 2020; Huang et al., 2020). Fortunately, some cases are resolved by just oxygen supply, but many individuals could not survive or require intensive care and end up on ventilation commonly (Xu Z. et al., 2020; Huang et al., 2020; Wadman et al., 2020). Furthermore, the development of pulmonary lymphopenia and increased neutrophil-lymphocyte ratio in almost 80 percent of infected patients is considered due to immune cells' stimulation toward the infection site (Tay et al., 2020a; Jamilloux et al., 2020). Lymphopenia is described as a result of either T cells’ death due to direct viral attack, cytokine-induced apoptosis of T cells, or immune cell redistribution (Jamilloux et al., 2020).

The clinicians believe that releasing a high amount of chemical signaling molecules or cytokine storm by the immune system may overreact or start attacking healthy cells and responsible for severe infection (Tay et al., 2020a; Wang et al., 2020b).

#### Cardiovascular System

Heart and blood vessels are rich in ACE2 receptors (Zheng et al., 2020; Chris Baraniuk, 2020). The studies conducted in China reveal that almost 20 to 44 COVID-19 percent of patients develop cardiac symptoms. These symptoms include arrhythmia, cardiac muscle damage, cardiac swelling and scarring, decreased heart function, and heart attack. Moreover, cardiac symptoms may develop secondary to pneumonia (Zhou F. et al., 2020; Ky and Mann, 2020; Wadman et al., 2020; Zheng et al., 2020). The cardiac symptoms may result in clotting defects, which are the additive COVID-19 severity and mortality. The hypercoagulation state is characterized by extended prothrombin time, high D-dimer levels, and fibrinogen with almost satisfactory partial thromboplastin time (Tang et al., 2020b; Merad and Martin, 2020). It is considered that cytokines, particularly IL-6, and endothelial cell injury are responsible for the activation of the coagulation system and suppression of the fibrinolytic system (Merad and Martin, 2020; Tang et al., 2020b; Wang et al., 2020b). It has been reported that almost 71.4 percent of nonsurvivors developed disseminated intravascular coagulation (DIC) (Tang et al., 2020b). The clots may progress to thrombosis formation, which may cause pulmonary embolism or stroke. It is considered to be a major cause of death due to COVID-19 infection (Zheng et al., 2020; Merad and Martin, 2020; Wang et al., 2020b). The data from the United States show that almost one-third of hospitalized patients had preexisting cardiovascular symptoms or diabetes. The studies suggest that the lack of oxygen and cytokine storm after the viral attack on the lungs is also responsible for blood vessels and heart damage (Tay et al., 2020a; Ky and Mann, 2020).

#### Renal System

Like other organs, kidneys are also abundant in ACE2 receptors (Chris Baraniuk, 2020, April 29). The studies show that about 59 percent of hospitalized patients develop proteinuria, whereas hematuria, increased blood urea nitrogen, and high levels of creatinine have been observed in 44 percent, 14 percent, and 10 percent of patients, respectively. Acute kidney injury (AKI) and kidney failure are seen as common (Li Z. et al., 2020). Reduced blood flow to the kidney is observed due to cytokine storms, resulting in kidney injury (Cheng et al., 2020). AKI is considered to be serious organ damage caused by COVID-19. A critical high serum creatinine (SCr) level and low urine output are reported during kidney injury (Xu D. et al., 2020). On the contrary, a study conducted in China by Wang et al. indicated fewer associations between AKI and COVID-19 (Wang et al., 2020a). Moreover, ventilators and some antivirals suggested for the treatment of COVID-19 may damage kidneys extensively, particularly in patients with preexisting conditions like diabetes, hypertension, and kidney diseases, respectively (Wadman et al., 2020) (Li Z. et al., 2020).

#### Central Nervous System

A large number of ACE2 receptors are present in the neural cortex and brain stem (Wadman et al., 2020; Chris Baraniuk, 2020, April 29). The SARS-CoV-2 has been detected in the cerebrospinal fluid. Neurological symptoms are seen in almost 5–10 percent of hospitalized patients (Wadman et al., 2020). However, the brain and nervous system damage should not be underestimated in patients on ventilators (Stevens et al., 2008). Hyperactivity of the nervous system, unconsciousness, loss of sense of smell and taste, meningitis, encephalitis, stroke, brain injury and seizure have been reported. Moreover, it is revealed that the cytokine storm and thrombosis are also responsible for brain swelling, stroke, and severe brain injury (Wu Y. et al., 2020).

#### Gastrointestinal Tract

The SARS-CoV-2 attacks the lining of the lower digestive tract that is rich in ACE2 receptors (Wadman et al., 2020; Chris Baraniuk, 2020). The virus has been detected in the stool samples of almost 53 percent of patients suffering from COVID-19. Additionally, the viral protein shell is found in the intestines biopsy indicating its replication in the gut linings (Zhou F. et al., 2020). Likewise, viral RNA has been detected on rectal swabs even after negative nasopharyngeal testing (Wang W. et al., 2020). The gastrointestinal symptoms include diarrhea, vomiting, and abdominal pain and have been observed in almost 20 percent of infected patients (Huang et al., 2020).

#### Liver

The injury to the liver and bile is found common in hospitalized patients, but the direct invasion of SARS-CoV-2 to the liver is not confirmed (Gu et al., 2020; Wadman et al., 2020). However, multiple events during the infection and administration of several drugs are considered responsible for elevated levels of liver enzymes and liver damage (Bangash et al., 2020). In a study conducted in China, 58% of 148 COVID-19 patients had an abnormal liver function. Higher levels of procalcitonin and C-reactive proteins have also been observed in these patients (Fan et al., 2020). Liver dysfunction is observed dominantly in severe cases (Zhang C. et al., 2020).

#### Eyes

Symptoms like conjunctivitis or pink eyes and watery eyes have been observed in almost one-third of hospitalized patients (Wadman et al., 2020). Chemosis, conjunctival hyperemia, epiphora, and increased secretions are reported in patients in addition to conjunctivitis. Patients with ocular symptoms have demonstrated extraordinary WBCs, neutrophil counts, procalcitonin, C-reactive protein, and lactate dehydrogenase levels compared to those without any ocular symptoms. Furthermore, RT-PCR assay of 90% of infected ocular patients showed positive results for SARS-CoV-2 from a conjunctival swab in addition to a nasopharyngeal swab (Wu P. et al., 2020).

#### Skin

The skin-related symptoms associated with COVID-19 were first reported in China, followed by Italy and in Spain; when a study was conducted on 375 patients with skin lesions and positive SARS-CoV-2 test, a relationship between skin lesions and COVID-19 was established (Diotallevi et al., 2020). It has been observed that the viral attacks on the ACE2 receptors, present in arterial and venous endothelial cells and arterial smooth muscle cells, trigger the host’s inflammatory response, including activation of mast cells and basophils, which may cause multiple skin conditions like rashes, diffuse or disseminated erythema, urticaria, livedo racemosa, blue toe syndrome, retiform purpura, vesicle trunk, purpuric exanthema, atopic dermatitis, and neutrophilic dermatoses, as well as less frequent cases of chilblains affecting fingers or toes (acral rash). It has been suggested that the skin manifestations may be the result of minor thrombotic events or damage to the endothelial walls of small distal vessels. These conditions have been observed in COVID-19 patients of all ages, but the rashes may be paraviral due to cytokines or drug-related during the treatment of any disease (Criado et al., 2020). Although these conditions may not be accompanied by pain or itching or other systemic symptoms, identifying rashes is important in earlier COVID-19 cases, and therefore attention to skin involvement during COVID-19 is also suggested (Bataille et al., 2020).

## Drugs Being Investigated for the Treatment of COVID-19 and Its Management

### Supportive Therapy

Antipyretics or NSAIDs for reducing fever and pain (Wu A. et al., 2020), oxygen therapy to maintain oxygen saturation (Røsjø et al., 2011), antibiotics as an empiric therapy (Rhodes et al., 2017), intravenous fluid resuscitation or vasopressor for regulating persistent shock (Schultz et al., 2017), early blood purification for reduction of renal workload and renal function recovery (Wang D. et al., 2020), beta-agonists such as dobutamine for the management of cardiac shock or failure, and systemic steroids against COPD exacerbation are commonly suggested as supportive therapy in COVID-19 infection (Alhazzani et al., 2020; World Health Organization, 2020a; Mcintosh, 2020). Furthermore, the use of vitamins as an immunity booster and some Chinese medicines against inflammatory responses has also been reported for the management of COVID-19 (People’s daily of China, 2020; Runfeng et al., 2020; Wang L.-S. et al., 2020). A description of such medications is as follows.

#### Antibacterials

##### Azithromycin

Clinical trials are currently perceiving its effectiveness against SARS-CoV-2 (Akram et al., 2020; Hinks et al., 2020; Sivapalan et al., 2020). In a trial conducted in France, it revealed that the group of COVID-19 patients who were receiving hydroxychloroquine along with azithromycin showed significant response in comparison with the group receiving hydroxychloroquine alone. Azithromycin was used in a dose of 500 mg per day on day one, followed by 250 mg per day for 5 days along with 600 mg hydroxychloroquine per day, respectively (Gautret et al., 2020). Another study showed its combination with other drugs; especially, hydroxychloroquine was beneficial on laboratory-confirmed COVID-19 patients (Sekhavati et al., 2020). However, Molina et al. and Remo H M Furtado et al. observed contrary results in their trials and did not support this combination therapy especially in patients with severe COVID-19 infection (Furtado et al., 2020; Molina et al., 2020). Similarly, the cardiac toxicity related to these drugs is also considered a major weakness of the regimen (Juurlink, 2020).

##### Teicoplanin

Teicoplanin has been shown to be active against the Ebola virus, SARS-CoV-1, and MERS-CoV and suggested for the treatment of COVID-19. It targets viral S protein and has been observed to be useful during the early phases of COVID-19 infection (Zhang J. et al., 2020). The recommended dose is 100–400 mg twice daily for 10 days (Parente and Laplante, 2017).

#### Immunomodulators and Immunosuppressants

##### Interferon-1

Clinical trials are being conducted to investigate its effectiveness against COVID-19 infection (Alavi Darazam et al., 2020). It has also been found to be effective previously against SARS-CoV-1 and MERS-CoV and suggested presently for the treatment of SARS-CoV-2 (Arabi et al., 2020; Jamilloux et al., 2020; Sallard et al., 2020). Researchers believed that SARS-CoV-2 could be more sensitive to IFN (Sallard et al., 2020). However, it has been advised for the treatment of COVID-19 patients who are suffering from hyperinflammation and ARDS (Jamilloux et al., 2020). The recently published interim results of the WHO SOLIDARITY trial consortium has downplayed the role of interferon alone or in combination with lopinavir (initially) to reduce the overall mortality of hospitalized moderate and severe COVID-19 patients. A total of over 1,412 patients reportedly enrolled in the study were compared with 4,088 patients with no study drug. The study has thus concluded that no difference has been observed in the 28-day survival rate among hospitalized patients receiving 44 µg subcutaneous injection thrice weekly or 10 µg daily for 6 days in patients on high oxygen or ECMO (Pan H. et al., 2020).

##### Systemic Corticosteroids

Methylprednisolone has been studied on COVID-19 patients with COPD and a dose of 1–2 mg per kg per day intravenously for 5–7 days has been found effective in reducing the mortality rate reported (Wu C. et al., 2020). Other studies reported 40–80 mg dose for a period of 3–6 days for the treatment of COVID-19 (Wu R. et al., 2020). Similarly, reduction in the disease course and improvement in symptoms have been observed in patients while administering corticosteroids (Wang Y. et al., 2020). However, the IDSA (Infectious Disease Society of American) recommends using the therapy only in the treatment of ARDS (Bhimraj et al., 2020).

A comprehensive trial in the United Kingdom named “Randomized Evaluation of COVID-19 Therapy” (RECOVERY), which is studying lopinavir-ritonavir, low-dose dexamethasone, hydroxychloroquine, azithromycin, tocilizumab, and convalescent plasma as potential treatments for the ongoing pandemic patients, has announced that low-dose dexamethasone (6 mg once daily po/iv) is found to have substantially (41%, patients on ventilation; 25%, patients of oxygen; 13%, no respiratory intervention) reduced the 28-day mortality of patients. The trial includes over 2,104 patients receiving low-dose dexamethasone and 4,321 patients on randomized to usual care (Peter Horby, 2020). FDA has also included it in the list of drugs for temporary compounding by outsourcing facilities and pharmacy compounders (FDA, 2020a). Some clinical trials are also being conducted to evaluate its effectiveness against COVID-19 (Maskin et al., 2020; Tomazini et al., 2020).

##### Tocilizumab/Sarilumab

Clinical trials are being conducted using these antibodies against SARS-CoV-2 (ClinicalTrials.gov, 2020; Farias et al., 2020). A study conducted in China revealed recovery of 20 out of 21 patients while using tocilizumab (NIH - U.S. National Library of Medicine, 2020, April 28). It has been found to be more effective in COVID-19 patients without the need for ventilator support and almost no toxicity is reported (Perrone et al., 2020). Furthermore, its efficacy has also been studied in the combination of high-dose methylprednisolone in COVID-19 patients and a decrease in mortality rate and mechanical ventilation support and increase in recovery have been observed (Ramiro et al., 2020). Similar significant results against COVID-19 infection and a reduction in fever at the first dose have also been reported in studies (Xu X. et al., 2020; Fu et al., 2020).

##### Sirolimus

It has been used for the treatment of viral infections, including infections caused by coronaviruses (Wang et al., 2014; Kindrachuk et al., 2015), and has been proposed as a potential candidate for the treatment of COVID-19 (Zhou Y. et al., 2020). Clinical trials are scheduled to be conducted using sirolimus in COVID-19 patients (NIH - U.S. National Library of Medicine, 2020, May).

#### Miscellaneous

##### NSAIDs

NSAIDs, especially acetaminophen, are considered important agents for the suppression of fever during an infection. These drugs also play a key role in reducing severe immune responses and preventing viral shedding. However, the side-effects such as GI bleeding, fluid retention, and kidney dysfunction related to these agents are of great concern (Little, 2020). Moreover, it has been claimed that NSAIDs, especially ibuprofen, upregulate ACE2 receptors and could exacerbate the factors for the COVID-19 infection (Day, 2020). Studies reported that no sufficient evidence had been found against the use of ibuprofen in patients with COVID-19 (Sodhi and Etminan, 2020).

##### Thiazolidinediones, ARBs, and ACE2 Receptor Blocker

It is believed that thiazolidinediones might be responsible for increased ACE2 receptor expression and could result in severe or deadly COVID-19 infection (Bauer et al., 2010; Fang et al., 2020). Researchers hypothesized that ARB's (Angiotensin Receptor Blocker) long-term use and ACE2 receptor blockers could result in overexpression of their receptors. It has been further concluded that high mortality in patients with a history of preexisting cardiovascular diseases and diabetes might be related to the long-term use of these drugs (Fang et al., 2020). On the contrary, a group of researchers claimed that no such evidence is available against thiazolidinediones, ARB, ACE inhibitors, or other related drugs (Gracia-Ramos, 2020).

##### Vasodilators (Nitric Oxide and Epoprostenol)

Since hypoxemia is a major risk of death in severe cases of COVID-19, vasodilators are considered to be useful. Unfortunately, no major study is reported on the treatment of vasodilators against SARS-CoV-2. However, the agents were used effectively against SARS-CoV-1 and MERS-CoV (Chen et al., 2004; Åkerström et al., 2005), and trials are also being proceeded or planned for the prevention and treatment of COVID-19 (Begun et al., 2020). In addition, FDA granted emergency expanded access to a biotherapeutics company, Bellerophon Therapeutics, for its inhaled nitric oxide (iNO plus) (GlobeNewswire, 2020, March 20). The company recently announced the first successful treatment of the COVID-19 patients (Bellerophon Therapeutics, 2020).

##### Vitamins

It is observed that the vitamin D levels decrease in several healthy individuals, especially during winter and additionally who get less exposure to sunlight, housebound, or work at night (Enwemeka et al., 2020). An adequate vitamin D level during summer strengthens the immune system and thereby decreases viruses’ attack (News Scientist, 2020, April 1). Clinicians claimed that the low level of vitamin D in the body could be the cause of the COVID-19 outbreak during the winter season (Enwemeka et al., 2020). Vitamin C, along with vitamin D and vitamin E, is also recommended in some studies. These vitamins have been beneficial in preventing respiratory infections and enhancing body resistance toward nCoV (Wang L.-S. et al., 2020).

##### Anticoagulants (Low-Molecular-Weight Heparin)

A decrease in mortality has been observed in patients while administering anticoagulants (Tang et al., 2020a). Low-molecular-weight heparin is suggested for the treatment of hypercoagulation and thrombosis-associated vascular damage in COVID-19 patients (Tang et al., 2020b). In addition, heparin has also been claimed to have anti-inflammatory and antiviral activities against SARS-CoV-2 (Shi C. et al., 2020; Mycroft-West et al., 2020). The European Society of Cardiology has recently schemed an anticoagulation protocol for coagulopathy management in patients suffering from COVID-19. Patients with respiratory rate >24 bpm, dyspnea, oxygen saturation <90%, rising D-dimer levels, elevated C-reactive protein, and elevated fibrinogen levels are characterized in high thrombotic risk group and various anticoagulation strategies are therefore suggested for them. A target of 60–85 prothrombin time (aPTT) range is considered and parenteral drip of heparin for patients admitted to Intensive Care Unit (ICU) or subcutaneous enoxaparin at a dose of 1 mg/kg two times a day for patients who do not require intensive care is suggested. Furthermore, Point-of-Care Ultrasound (POCUS) is recommended for deciding the continuation of the anticoagulation therapy (Atallah et al., 2020).

##### Traditional Herbal Medicines

Traditional herbal medicines have been found useful for the treatment of epidemic outbreaks, including influenza and coronaviruses (Chen et al., 2011; Xiaoyan et al., 2018; Redeploying plant defences, 2020; Xiaoyan et al., 2020). Traditional medicine treatment guidelines have also been issued by China and Korea on the treatment of COVID-19 (Ang et al., 2020). Chinese medicines, particularly Shuanghuanglian oral liquid and LianhuaQingwen capsule, have been extensively used for the treatment of COVID-19 disease (People's daily of China, 2020; Runfeng et al., 2020). The other traditional herbal medicines commonly used for the treatment of COVID-19 include *Astragalus membranaceus, Saposhnikoviae divaricata*, *Glycyrrhiza uralensis*, *Rhizoma atractylodis macrocephalae*, *Fructus forsythia, Lonicerae Japonicae Flos*, *Atractylodis Rhizoma*, *Radix platycodonis*, and *Agastache rugosa* (Luo et al., 2020). It is believed that these medicines could play a key role in the reduction of inflammatory responses developed in the human body as a result of viruses and bacteria (People's daily of China, 2020; Runfeng et al., 2020)

### Specific Therapy

It is believed that the drugs targeting the SARS-CoV-2 main protease (M^pro^), an enzyme that is important to viral replication and transcription, could play a key role in COVID-19 treatment (Jin Z. et al., 2020). A list of drugs is described as follows.

#### Antiprotozoals

##### Chloroquine (CQ) and Hydroxychloroquine (HCQ)

Researchers analyzed that antiprotozoals reduce cytokine storm, the main cause of severe infection and death in COVID-19 (Cao, 2020). The therapy is claimed to be responsible for the inhibition of ACE2 receptors glycosylation. The drugs also bind with viral S protein, resulting in the prevention of SARS-CoV-2 entry into the cells (Savarino et al., 2006; Liu J. et al., 2020; Wang M. et al., 2020). *In vitro* studies have shown supportive results in reducing viral replication and, thus, symptoms duration (Gao et al., 2020; Singh et al., 2020). A study conducted on patients with positive SARS-CoV-2 indicated that the viral RNA became undetectable on day 6 after administering 200 mg HCQ three times a day (Frie and Gbinigie, 2020).

On March 28, both were granted EUA (Emergency Use Authorization) for the treatment of COVID-19 by FDA (FDA, 2020, March 28). Later FDA, on April 24, warned the use of these agents, alone or in combination with other drugs, particularly azithromycin, outside a hospital setting because of the reported evidence of abnormal heart rhythm in a clinical trial (FDA, 2020, April 24) (Borba et al., 2020). Furthermore, their use against COVID-19 was not successful and therefore not supported by WHO (WHO, 2020, March 11). HCQ is considered less toxic due to the hydroxyl group present in its structure, which helps in easy clearance from the body (Schultz and Gilman, 1997; Singh et al., 2020).

In rather recent developments, the large-scale trials have either stopped or paused the study on HCQ, citing that the treatment does not offer any improvement in mortality; moreover, initial data showed significant adverse effects associated. This is true for RECOVERY and SOLIDARITY trials as well, which are funded by UKRI and WHO, respectively. In the case of the RECOVERY trial, 1,542 patients treated with HCQ had higher (25.7%) fatality in 28 days compared to 3,132 patients (23.5%) with standard care. Similarly, SOLIDARITY trials by WHO were also suspended on May 24, 2020, owing to reports of toxicity and nonsuperiority of the treatment. The development has also led to the retraction of a major article published in Lancet where the journal could not verify the data due to confidentiality issues of the patients amid the widespread skepticism of the drug (Kupferschmidt, 2020). Similar results have also been observed recently by Lyngbakken et al., and no improvement was found for using 400 mg HCQ two times a day for 7 days in hospitalized patients suffering from COVID-19 infection (Lyngbakken et al., 2020).

##### Nitazoxanide

Nitazoxanide is suggested to have strong antiviral activity against SARS-CoV-2 (Srivatsan Padmanabhan and Tech, 2020). Additionally, it has also been proposed in combination with hydroxychloroquine for the treatment of COVID-19 infection and suggested to help eradicate viral load and control overwhelming the immune system, particularly in severe cases (Srivatsan Padmanabhan, 2020). The optimal doses against SARS-CoV-2 are predicted to be 1,200 mg four times, 1,600 mg three times, and 2,900 mg two times a day in a fasted state and 700 mg four times, 900 mg three times, and 1,400 mg two times a day in fed state, respectively (Rajoli et al., 2020). In a recent clinical trial conducted on ambulatory, hospitalized, and pregnant women suffering from COVID-19 infection, nitazoxanide has been observed to be a safe therapy and found effective against SARS-CoV-2 (Meneses Calderón et al., 2020).

#### Antivirals

##### Remdesivir

It has been widely used against the Ebola virus and has shown effective against other single-stranded RNA viruses such as Marburg virus, Nipah virus, parainfluenza type 3 virus, and human coronaviruses (Warren et al., 2016; Lo et al., 2017). Clinical trials are being carried out currently to evaluate its safety and efficacy against SARS-CoV-2 (Al-Tawfiq et al., 2020; NIH - U.S. National Library of Medicine, 2020a; NIH - U.S. National Library of Medicine, 2020b). Its intravenous administration to COVID-19 patients resulted in notable recovery from pneumonia (Holshue et al., 2020). A study in USA, Japan, and Europe or Canada showed clinical improvements in 36 out of the 53 hospitalized severe COVID-19 patients (68%) with remdesivir at a dose of 200 mg on day 1, followed by 100 mg per day for 9 days (total: 10 days of therapy). In terms of therapeutic goals, in a large randomized, double-blind, placebo-controlled clinical trial conducted in over 1,000 patients, remdesivir was found to have shortened the duration of therapy from 15 to 11 days and improvements in mortality rates have been observed (Beigel et al., 2020). In May 2020, FDA issued EUA to this drug for the treatment of severe COVID-19 cases in both adults and children. The FDA further defined severe conditions as individuals with low blood oxygen saturation levels or requiring mechanical oxygen support (FDA, 2020, May 1). Later, in October 2020, it became the first antiviral drug approved by the FDA for use in COVID-19 patients above 11 years of age and 40 kg individuals (FDA, 2020 ; U.S.F.D.A., 2020). However, very recently, the use of remdesivir has been rejected by WHO after the interim results of the SOLIDARITY trial are released. A total of 2750 COVID-19 patients were given 200 mg loading dose followed by 100 mg of drug once daily till the 9th day. The data showed that the treatment group did not show any improvement over the no-drug-of-study group comprising 4,088 patients. The drug failed to improve overall mortality or prolonged the initiation of ventilation of moderately ill patients. (Pan H. et al., 2020). Though the trial is a multicenter global study, its case-by-case recommendation remains largely weak against low-risk vs. high-risk patients as described in randomized double-blind placebo-controlled clinical study “adaptive COVID-19 treatment trial” (ACTT-1), which favors the treatment with the drug and was subsequently used by FDA before giving approval. The final results, however, are awaited before any conclusion is made on its (SOLIDARITY trial) effectiveness in any subgroups of patients (NIAID, 2020; Pan H. et al., 2020).

##### Lopinavir/Ritonavir

Clinical trials have been investigated using a combination of a dose of 400 mg for lopinavir and 100 mg for ritonavir two times a day for the treatment of COVID-19. The clinicians claimed that no remarkable benefits were observed. The studies further found nausea, diarrhea, and asthenia as common side-effects (Cao et al., 2020). However, a study conducted in China using 400 mg lopinavir per day with or without IFN- α2b has claimed that it is effective against COVID-19. But, the consideration of gastrointestinal side-effects and hypokalemia was also suggested (Liu F. et al., 2020). Similarly, improved COVID-19 related clinical symptoms such as fever and no reduction in SARS-CoV-2 titers have been reported in a study conducted in Korea (Lim et al., 2020). Another clinical trial has also been announced recently by Prasan Kumar Panda et al. for investigating the efficacy of lopinavir-ritonavir or hydroxychloroquine along with ribavirin in hospitalized COVID-19 patients (Panda et al., 2020). Similar to the case of remdesivir, lopinavir and ritonavir have also failed to demonstrate any considerable superiority to other treatment options used for patients with moderately ill patients in the SOLIDARITY trials (Pan H. et al., 2020).

##### Ribavirin

Ribavirin has been observed to be effective, particularly in combination with IFN, against viruses, including coronaviruses (Scott and Perry, 2002; Khalili et al., 2020). However, a decrease in hemoglobin concentration is reported in patients with COVID-19, while administering this drug (Khalili et al., 2020). Its efficacy in hospitalized COVID-19 patients has been recently investigated alone and in combination with sofosbuvir/daclatasvir and no significant improvement was observed. However, clinicians suggested further investigation on large clinical trials (Abbaspour Kasgari et al., 2020; Eslami et al., 2020).

##### Umifenovir

It has been reported effective against both SARS-CoV-1 and SARS-CoV-2 (Blaising et al., 2014; Dong et al., 2020). A retrospective study found that an increase in efficacy of lopinavir and ritonavir was observed against SARS-CoV-2 when augmented with umifenovir (Deng et al., 2020).

##### Favipiravir

It has been proposed as an experimental drug for the treatment of COVID-19 infection (Li and De Clercq, 2020). The clinical trial indicated that patients suffering from moderate COVID-19 infection when treated with favipiravir showed remarkable recovery within a week. It has also been found clinically superior to umifenovir against SARS-CoV-2. The former showed about 71% recovery rate, and the latter 55% only (Chen C. et al., 2020). Several clinical trials are being conducted to examine its safety and efficacy against SARS-CoV-2 (Seneviratne et al., 2020; WHO, 2020, April 11). It has been observed to be effective against SARS-CoV-2 at a dose of 1600 mg twice a day first, followed by 600 mg two times a day for 13 days (Cai et al., 2020). However, low blood concentration of this drug in severe COVID-19 patients compared to healthy individuals has been observed in a recent clinical trial and therefore, further investigation for the development of optimal treatment strategy in critically ill COVID-19 patients has been suggested (Irie et al., 2020).

##### Oseltamivir

Clinical trials are being conducted using this drug alone and in combination with other antivirals and antiprotozoals against COVID-19 infection (Rosa and Santos, 2020). However, scientists claim that no satisfactory results have been observed while administering oseltamivir to COVID-19 patients (Wu A. et al., 2020). Similarly, it has been suggested that as SARS-CoV-2 does not contain neuraminidase enzyme, oseltamivir and other related drugs are not expected to be effective against COVID-19 (Orders, 2020).

##### Convalescent Plasma Therapy

**FIGURE 4:**
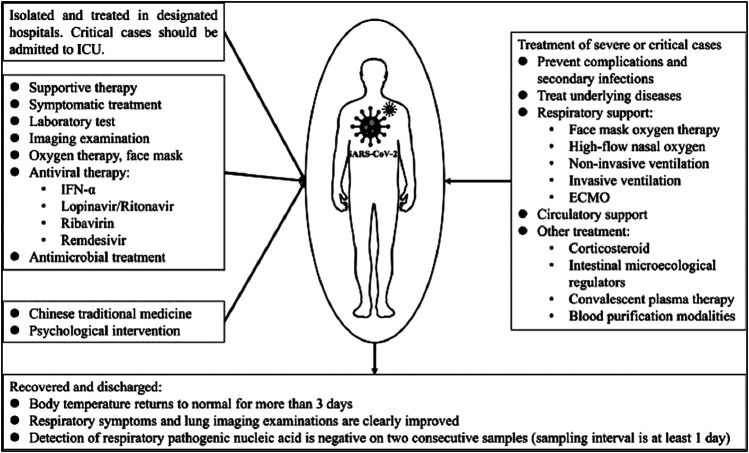
The treatment and management option for COVID-19 patients [reproduced with permission] (Yang Y. et al., 2020).

Convalescent plasma therapy has been successfully used previously against SARS-CoV and MERS-CoV coronaviruses (Zhang et al., 2005; Mair-Jenkins et al., 2015) and is now being explored against COVID-19 (Shen C. et al., 2020; Chen L. et al., 2020). The technique has also been recommended as an emergency investigational new drug application by the FDA for the treatment of fatal or deadly COVID-19 infections (Food and Administration, 2020). A number of clinical trials exploring the safety of the therapy have concluded that the use of convalescent plasma is safe with or without other treatment options used. However, the studies have largely failed to confirm its effectiveness in a variety of settings, especially in a large randomized clinical trial with over 100 enrolled patients; the effectiveness of the therapy has been largely inconclusive in improving the condition of moderately and severely ill patients (Agarwal et al., 2020; Li L. et al., 2020; Olivares-Gazca et al., 2020). Moreover, in one study, the convalescent plasma transfusion failed to increase the neutralizing antibody titers in recipients (Bradfute et al., 2020). The treatment and management option is also described by Yang et al in 2020 (see Figure 4)

### Landscape of Vaccine Development for SARS-CoV-2

In the wake of the current pandemic, an unprecedented response has been observed globally on the front for vaccine development against SARS-CoV-2 to protect the large masses of the global population from getting infected with long-term immunity against the virus. There are largely three ways due to which this development response is unprecedented in the history of pandemics ever. The first reason is that there are over 120 vaccine research groups and teams participating in the development, comprising majorly large and small commercial sector biotech companies followed by academic institutions and public sector research organizations (Shang et al., 2020; WHO, 2020b). A large fraction of these teams are working on already developed and tested platforms such as live-attenuated virus and chemically inactivated viruses that have been successful for a wide range of viral diseases (Shang et al., 2020). This also ensures that if one method becomes successful, the challenges to develop and scale things up would not be cumbersome. Moreover, novel strategies like DNA plasmids and mRNA-based vaccines are also being touted as a potential breakthrough in the field as these platforms offer high production and quality control during manufacturing and can be replicated to suffice the global demand (Callaway, 2020b). On the other hand, some leading researchers are skeptical of the timeline prediction of 18 months for the vaccine citing various technical challenges and regulatory oversight reasons (GeneScript, 2020). The fastest time for a vaccine development that has been recorded is of 7 months for the Ebola, Swine flu, and Zika virus. However, in the case of SARS-CoV-2, it took only 10 weeks for RNA-based Moderna vaccine candidate to enter clinical trials (Callaway, 2020a; GeneScript, 2020). Concise information about a variety of vaccine platforms currently under various stages of clinical development (regulatory approval for human use) is given in Table 1. Moreover, the detailed landscape of vaccines that successfully demonstrated a safety profile and immunogenicity in phase I and II studies has been given in Tables 2 and 3. A variety of platforms are being explored for the development of a vaccine. These include mRNA-based vaccines, DNA- and plasmid-based vaccines, nonreplicating viral vectors, and attenuated viral vaccines, protein subunits. Apart from that, some already existing vaccines are also being explored for likely cross-/nonspecific immunity against SARS-CoV-2 infection. These vaccines include BCG, polio, and MMR vaccines, which are also part of many national child-immunization programs in the developing countries. Among all the vaccines being developed, ten vaccines have entered phase III clinical trials and have shown promising results in terms of safety and immunogenicity (WHO, 2020b).

**TABLE 1:** Number and types of SARS-Cov-2 vaccines with respect to clinical development phase [sources: ClinicalTrials.gov, WHO COVID-19 (DRAFT landscape of COVID-19 candidate vaccines), and Biorender.com (COVID-19 vaccine tracker)].

Types of vaccines	Clinical phase of development*	No. of vaccines
Repurposed	III/IV	3
	III	1
	II	1
RNA-based vaccines	I/II/III	2
	I/II	3
	I	2
Nonreplicating viral vector vaccines	I/II/III	4
	I/II	Nil
	I	3
DNA/plasmid vaccines	I/II/III	Nil
	I/II	4
	I	2
SARS-CoV-2 inactivated viral vaccine	I/II/III	3
	I/II	4
	I	Nil
Protein subunit/peptide vaccines	I/II/III	1
	I/II	4
	I	8
Modified antigen-presenting cells–based immunization vaccine	I/II	2
	I	1
Replicating viral vector vaccine	I/II	1
	I	4
Others (virus-like particles)	I	2

^*^ Objective of clinical phase of vaccine development: Phase I (safety and immunogenicity), Phase II (safety, immunogenicity, and potential efficacy), Phase III (large-scale efficacy and evaluation of toxicity and immunogenicity).

**TABLE 2:** Description of vaccines under the clinical phase of development for SARS-CoV-2 (repurposed and RNA vaccines) [sources: ClinicalTrials.gov, WHO COVID-19 (DRAFT landscape of COVID-19 candidate vaccines), and Biorender.com (COVID-19 vaccine tracker)].

Type	Name of vaccine	Description	Primary developer/sponsor	Purpose	Phase of clinical development (no. of trials) [no. of participants]
Repurposed vaccine	BCG	Live-attenuated bacterial *Bacillus* Calmette-Guerin (BCG) vaccine (nonspecific immunity)	Multisite trials with multiple sponsors^a^	Treatment and prevention	Phase IV (4) [1,000 + 900 + 1,800 + 5,200] Phase III (15) [1,500 + 1,900 + 2,100 + 2,175 + 908 + 3,626 + 2,038 + 59 + 1,120 + 1,500 + 1,000 + 500 + 900 + 10,078 + 1,200]
	OPV	Oral polio vaccine (nonspecific immunity)	Bandim Health Project NeuroActiva, Ltd. Biomed Industries, Ltd.	Prevention	Phase IV (1) [3,400] Phase III (1) [3,600]
	MMR	Live-attenuated viral measles-mumps-rubella (MMR) vaccine (nonspecific immunity)	Kasr El Aini Hospital, Egypt	Prevention	Phase III (2) [200 + 60]
	IMM-101	Heat-killed *Mycobacterium obuense* (nonspecific immunity)	Canadian Cancer Trial Groups, Immodulon Therapeutics, Ltd., BioCan Rx	Treatment	Phase III (1) [1,500]
RNA-based vaccines	nCoV mRNA-1273	Lipid nanoparticles dispersion containing mRNA encoding for SARS-CoV-2 spike protein	Moderna TX, Inc./National Institute of Allergy and Infectious Diseases (NIAID)	Prevention	Phase III (1) [30,000] Phase II (1) [600] Phase I (1) [155]
	BNT162 (a1, b1, b2, c2)	Four lipid nanoparticles encapsulated RNA-based vaccines: 2 nucleoside modified RNA (modRNA), 1 uridine containing mRNA (uRNA), and 1 self-amplifying mRNA (saRNA)	BioNTech RNA Pharmaceuticals GmbH and Pfizer, Inc. Fosun Pharma	Prevention	Phase II/III (1) [BNT162b1, b2: 43,998] Phase I/II (1) [BNT162b2: 160] Phase I/II (1) [BNT162b3: 120] Phase I/II (1) [BNT162b1: 144] Phase I/II (1) [BNT162a1, b1, b2, c2: 456]

^a^ University of Campinas, Brazil/UMC Utrecht/Radboud University, Netherlands/Universidad de Antioquia, Colombia/TASK Applied Science/Ain Shams University, Egypt/Murdoch Children’s Research Institute/Royal Children's Hospital, Australia/Bandim Health Project/University of Southern Denmark/Vakzine Projekt Management GmbH/FGK Clinical Research GmbH, Germany/Assistance Publique- Hôpitaux de Paris, France/ Conselho Nacional de Desenvolvimento Científico e Tecnológico, Instituto de Infectologia Emílio Ribas, Universidade Estadual de Campinas, Unicamp, Pontifícia Universidade Católica de Campinas, PUC-Campinas, Faculdade de Medicina de Ribeirão Preto/USP, Faculdade de Medicina de Botucatu, Unesp, Federal University of São Paulo, State Hospital Dr. Leandro Franceschini, Sumaré, Unicamp, Paulinia Municipal Hospital.

**TABLE 3:** Description of vaccines under clinical phase development for SARS-CoV-2 (viral vector, attenuated vaccines, and protein subunit vaccines) [sources: ClinicalTrials.gov, WHO COVID-19 (DRAFT landscape of COVID-19 candidate vaccines), and Biorender.com (COVID-19 vaccine tracker)].

Type	Name of vaccine	Description	Primary developer/sponsor	Purpose	Phase of clinical development (no. of trials) [no. of participants]
Nonreplicating viral vector vaccines	Ad26.COV2.S	Nonreplicating adenovirus type 26 expressing SARS-CoV-2 spike protein	Janssen Vaccines and Prevention Beth Israel Deaconess Medical Center Johnson and Johnson	Prevention	Phase III (2) [30,000 + 60,000] Phase I/II (1) [1,045] Phase I (1) [250]
	AZD1222 (ChAdOx1 nCoV-19)	Nonreplicating adenovirus type 5 expressing SARS-CoV-2 spike protein	Jenner Institute University of Oxford/AstraZeneca	Prevention	Phase III (3) [100 + 5,000 + 40,051] Phase II/III (1) [12,390] Phase I/II (3) [1,090 + 256 + 2,000]
	Ad5-nCoV	Nonreplicating adenoviral type 5 vector expressing SARS-CoV-2 spike protein	CanSino Biologics Inc./Canadian Center for Vaccinology/Institute of Biotechnology, PLA of China	Prevention	Phase III (3) [508 + 40,000 + 500] Phase II/III (2) [696 + 481] Phase I (2) [108 + 144]
	Gam-COVID-Vac-Lyo	Composite vaccine with two adenoviruses (Ad5 and Ad26) containing SARS-CoV-2 genes for spike protein	Gamaleya Research Institute of Epidemiology and Microbiology, Russia	Prevention	Phase III (2) [40,000 + 100] Phase I/II (2) [38 + 110]
SARS-CoV-2 inactivated viral vaccine	CoronaVac	Chemically inactivated SARS-CoV-2 viral vaccine	Sinovac R&D Co., Ltd., China Health Institutes of Turkey Butantan Institute PT Biopharma Faculty of Medicine Universitas Padjadjaran	Prevention	Phase III (4) [13,060 + 13,000 + 1,620 + 1,040] Phase I/II (2) [744 + 552] Phase I (1) [422]
	SARS-CoV-2 vaccine unnamed	Chemically inactivated SARS-CoV-2 viral vaccine	Wuhan Institute of Biological Products, Sinopharm Universidad Peruana Cayetano Heredia	Prevention	Phase III (1) [6,000] Phase I (1) [288] Phase II (1) [1,168]
	BBIBP-CoV	Chemically inactivated SARS-CoV-2 viral vaccine	Beijing Institute of Biological Products and Sinopharm	Prevention	Phase III (3) [45,000 + 3,000 + 15,000] Phase II (1) [1,648] Phase I (1) [480]
Protein/peptide vaccines	SARS-CoV-2 rS NVX-CoV2373	Adjuvant nanoparticles with conjugated spike proteins	Novavax Inc. Coalition for Epidemic Preparedness (CEPI) Department of Health and Human Services (US)	Prevention	Phase III (2) [9,000 + 30,000] Phase II (2) [4,400 + 1,419] Phase I (2) [131 + 1,419]

## Preventive Measures

As no specific treatment is available for this nCoV, it is recommended to obtain preventive measures and reduce the spread of the virus. It is advised to maintain personal hygiene, proper ventilation, a healthy lifestyle, and adequate nutritional consumption to boost immunity and enhance self-resistance (Yoshikawa and High, 2001; Simpson et al., 2015). Handwashing with soap and water or alcohol-based sanitizer after contact with any contaminated surface is directed (World Health Organization, 2020b). Protective equipment such as face mask, gown, gloves, face shield or goggles, and N95 respirator is suggested, especially in hospital settings (World Health Organization, 2020b). A study conducted in a hospital in Wuhan, China, on the association between the use of face masks and spread of COVID-19 has revealed that the infection rate in the departments using face masks, disinfectants, and handwashing was lower than that in the departments not using or frequently using the preventive measures (Wenjie et al., 2020). Close interaction is the cause of viral transmission (World Health Organization, 2020b). The infected people are recommended to be isolated or use airborne infection isolation (AIIR)/negative pressure isolation (NPI) room (Wax and Christian, 2020). Social distancing by reducing mass gatherings, social events, and group meetings is considered the best preventive measure (CDC, 2020, March 27).

## Conclusion

COVID-19 disease is an enormous challenge to the global community. This once-in-a-century pandemic has catastrophically affected our daily lives and is an unprecedented threat to the economies of many leading nations across the globe. Besides plethoric technological advancement and awareness, the world was not prepared to face the sudden outbreak of this disease. The pace of disease progression in different regions of the world has made it imperative that quick and honest preventive measures should be implemented at macro and micro levels to limit the spread till the development and approval of effective vaccine and evidence-based management at a global scale. The virus demands respect, so keeping self-hygiene, wearing masks, sanitizing, and maintaining social distancing seem to be the only effective means of mitigating the disease. Cultural diversity among different regions should be considered in developing robust strategies and communication. Many vaccines are under various phases of clinical trials, their efficacy, safety, scale-up production, supply chain management, and cold chain maintenance are another set of challenges, especially for developing and underdeveloped nations.
